# Stem Cell Therapy for Parkinson’s Disease: A Mechanistically Distinct Role for Muse Cells

**DOI:** 10.3390/jcm15114370

**Published:** 2026-06-05

**Authors:** Michael H. Mesches, Ann-Charlotte Granholm, Daniel Paredes, Karin Mesches, Yo Oguma, Mari Dezawa

**Affiliations:** 1 Department of Pediatrics, University of Colorado Anschutz Medical Campus, Aurora, CO 80045, USA; michael.mesches@cuanschutz.edu; 2 Department of Neurosurgery, University of Colorado Anschutz Medical Campus, Aurora, CO 80045, USA; 3 Knoebel Institute for Healthy Aging, University of Denver, Denver, CO 80208, USA; daniel.paredes@du.edu; 4 Department of Neurology, Johns Hopkins School of Medicine, Baltimore, MD 21205, USA; 5 SciTechEdit International LLC, Highlands Ranch, CO 80130, USA; karin.mesches@scitechedit.com; 6 Department of Stem Cell Biology and Histology, Tohoku University Graduate School of Medicine, Sendai 980-8575, Miyagi, Japan; yo.oguma.s7@dc.tohoku.ac.jp (Y.O.); mari.dezawa.e1@tohoku.ac.jp (M.D.)

**Keywords:** cell replacement therapy, Muse cells, Parkinson’s disease, pluripotent cells, stem cells

## Abstract

Cell replacement therapy is a promising investigational approach for Parkinson’s disease (PD), a neurodegenerative disorder characterized by progressive loss of dopaminergic neurons in the substantia nigra. Although current PD therapies provide symptomatic relief, none halt or reverse disease progression. Early transplantation studies using fetal dopaminergic neurons provided proof of concept for PD cell replacement, with recent efforts focusing on pluripotent stem cell-derived dopaminergic progenitors that are now entering clinical testing. These strategies face challenges, however, including immune compatibility, tumorigenic risk, and the need for controlled differentiation and functional integration. Multi-lineage differentiating stress-enduring (Muse) cells are endogenous, non-tumorigenic pluripotent-like stem cells that home to sites of tissue injury and differentiate in response to the host microenvironment. A targeted literature search of PubMed and Scopus, however, did not identify prior reviews specifically addressing Muse cells in the context of PD, highlighting a gap in the literature. Here, we examine current limitations of established cell-replacement approaches and consider whether Muse cells may represent a mechanistically distinct cell source. Early clinical studies of Muse cell therapy in stroke and amyotrophic lateral sclerosis suggest an encouraging safety profile and preliminary signals of potential therapeutic benefit, although these findings are based on small, early-stage trials and require confirmation. The evidence supporting Muse cell therapy in PD is currently limited to a single preclinical animal study, supported by mechanistic in vitro findings and indirect evidence from other neurologic disease models; therefore, its relevance to PD remains to be established, and current evidence is insufficient to support conclusions regarding clinical efficacy. Together, these observations provide a rationale for further targeted preclinical investigation and support the systematic evaluation of Muse cells as a mechanistically distinct candidate for regenerative therapy in PD.

## 1. Introduction

Parkinson’s disease (PD) is the second most common progressive neurologic disorder worldwide [1,2]. Motor symptoms of PD include tremor at rest, rigidity, bradykinesia, and postural instability [3]. Non-motor symptoms are variable and include neuropsychiatric manifestations (anxiety, apathy, depression, delusions), cognitive impairment and dementia, autonomic dysfunction (constipation, urinary incontinence, orthostatic hypotension), fatigue, and sleep disorders [4,5]. The biologic mechanisms underlying PD-related neurodegeneration remain under debate, but the accumulation of pathologic forms of α-synuclein in intracellular Lewy bodies [6,7] and chronic neuroinflammation in brain regions related to motor function are established findings [8]. Mitochondrial dysfunction, impaired protein clearance, and oxidative stress are also thought to contribute to neurodegeneration in PD [8]. Mutations in *LRRK2*, *PARK2*, *PARK7*, *PINK1*, and *SNCA* are linked to these multiple and parallel disease processes in the brain [4,9]. Environmental factors can also contribute to PD pathology [10]. Most scientists agree that PD is caused by a combination of environmental and genetic or epigenetic factors. The high level of heterogeneity in PD related to phenotypic, genetic, and clinical presentation complicates evaluation of novel therapies. In addition, disease duration, motor phenotype, baseline severity, cognitive status, age, and comorbidity burden may influence treatment response and complicate interpretation of benefits in regenerative-therapy studies [11]. Future clinical studies of cell-based therapies should therefore stratify or account for relevant baseline characteristics rather than assuming uniform treatment responsiveness across the PD population. Importantly, PD is a multisystem disorder with both motor and non-motor manifestations [5], and dopaminergic cell replacement alone may not fully address non-motor symptoms or broader disease pathology.

Given the challenges of disentangling the relative contributions of genetic, environmental, and epigenetic factors to PD pathogenesis, research has focused on developing novel and disease-modifying treatment options for this severe and progressive neurologic disease. The first drug developed for PD was 3,4-dihydroxy-l-phenylalanine (L-DOPA), a dopamine (DA) precursor [12] that is administered in combination with carbidopa to reduce peripheral side effects [5]. Improvement of symptoms following treatment with L-DOPA/carbidopa is a key diagnostic indicator of PD [12], a discovery that contributed to Dr. Arvid Carlsson being awarded the Nobel Prize in Physiology or Medicine in 2000 [13]. Although L-DOPA continues to be widely used, it does not alter disease progression and eventually leads to motor complications, including wearing-off effects and dyskinesia [14]. Other medications for PD, such as DA agonists or monoamine oxidase-B inhibitors, are aimed at reducing other neurologic or psychiatric symptoms of PD [15]. Monoamine oxidase-B inhibitors may be effective as monotherapy in early PD but are most often used as an adjuvant therapy with L-DOPA/carbidopa in later stages of the disease [15]. Supportive treatments to slow the progression of PD, such as diet, exercise, physical therapy, occupational therapy, and speech therapy, may extend the quality of life for patients with PD [5]. Although current medications and/or therapies can slow the progression of PD, these treatment options are not disease modifying in that they cannot replace lost or dysfunctional neurons. Therefore, the therapeutic potential of cell replacement for PD has been a major research focus over the last couple of decades.

Cell replacement therapies for PD began to be developed in the 1980s and 1990s, first in animal models and then in human patients. Early studies included autografts of adrenal medulla chromaffin cells (known to transform into DA-producing cells when grafted into the central nervous system (CNS), see, e.g., Kordower, et al. [16]) or allografts of fetal dopaminergic neurons [16,17,18,19,20,21]. The rationale was to replace the dopaminergic innervation lost in the striatum with DA-producing cells. Transplantation of human fetal ventral mesencephalic tissue provided early clinical proof of concept that dopaminergic cell replacement can improve motor symptoms in PD [22]. Graft-induced dyskinesia was a common severe side effect in PD patients receiving fetal dopaminergic neuron grafts [23]. Various reasons for these L-DOPA independent side effects have been described, including (a) aberrant transmitter release from serotonergic neurons in the raphe nucleus erroneously included in the grafted tissues, (b) excessive dopamine release from fetal neurons, and (c) uneven reinnervation of the recipient striatum. Hot spots in DA neurotransmission, presumably caused by young DA neurons innervating a more sensitive adult or aged recipient brain, were observed in postmortem tissue [24]. Although fetal dopaminergic cells robustly survived in grafts for up to 14 years post-transplantation [18], subsequent studies revealed that grafted cells eventually developed Lewy body pathology and degenerative features resembling the PD pathology of endogenous dopaminergic neurons [19,25]. Potential explanations for the emergence of pathology in grafted neurons over time include the propagation of α-synuclein in a prion-like manner [7,26], allowing pathology to spread from host tissue to grafted cells, or the vulnerability of transplanted fetal tissue to spontaneous generation of pathology within the host environment [27]. These long-term findings prompted investigators to evaluate alternative cell sources and strategies for intracranial cell replacement in PD, as discussed below. In addition to exploring new donor cell sources, approaches that enhance trophic support for surviving neurons have also been investigated. We recently demonstrated that autografting small segments of a patient’s sural nerve can stimulate surviving dopaminergic neurons and provide trophic support for several years following transplantation [28]. These findings support the autograft approach and suggest that effective cell-replacement strategies may benefit from growth-factor support for both endogenous degenerating neurons and transplanted cells.

In this narrative review, we examine current approaches to cell replacement therapy for PD and evaluate multi-lineage differentiating stress-enduring (Muse) cells as a mechanistically distinct and emerging regenerative platform. We distinguish between established and clinically advancing PD cell-replacement strategies and the earlier-stage evidence base for Muse cells, for which PD-specific data remain limited. A targeted literature search was conducted in PubMed and Scopus using “cell replacement therapy AND Parkinson’s disease AND stem cells.” Studies were selected based on relevance to PD cell-replacement strategies, Muse cell biology, and translational or clinical evidence in neurologic disease. As this is a narrative review, no formal risk-of-bias assessment was performed, and selection or citation bias cannot be excluded.

## 2. Stem Cell Therapy for PD

Although symptomatic treatments yield an initial reduction in clinical symptoms for PD patients that can last for years, these treatments eventually exhibit reduced efficacy and increased side effects [12], as mentioned above, making it necessary to develop therapies that modify disease progression rather than only providing symptomatic relief. The potential of stem cells as cell-replacement therapies has thus been actively investigated over the last three decades (see, e.g., Song, et al. [29], Parmar, et al. [30]). Research into stem cell therapy for PD has included various stem cell sources, including embryonic stem cells (ESCs), induced pluripotent stem cells (iPSCs), and mesenchymal stem/stromal cells (MSCs; Table 1) [29]. Early stem cell research was based on the assumption that pluripotent stem cells (PSCs) could be directed to differentiate into specific neuronal cell types, including dopaminergic neurons derived from ESC lines or fetal tissue grafts (see Table 1 and [31,32]). The use of ESCs and fetal-tissue-derived grafts raised practical and ethical concerns [33], however, prompting investigators to explore alternative sources of inducible or reprogrammable cells. While the stem cell types listed in Table 1 are considered to have strong potential for cell replacement in neurologic conditions, their developmental stages differ substantially, with pluripotent stem cell-derived dopaminergic progenitors currently representing the most advanced regenerative approach and entering early-phase clinical trials. Clinical translation remains in its early stages, and long-term, clinically meaningful benefits for PD patients have not yet been clearly demonstrated [34]. The success rate of stem cell treatment in PD may also depend on patient-specific factors, including age, comorbidities, and disease duration [32,34,35].

### 2.1. Embryonic Stem Cells

ESCs are pluripotent cells derived from the inner cell mass of the blastocyst and can self-renew and differentiate into any cell type in the body, including dopaminergic neurons, under the appropriate conditions [48,49]. While this cell source was among the earliest investigated for grafting in neurodegenerative diseases, clear and consistent long-term clinical benefit from ESC-derived grafts in patients with PD has not yet been definitively demonstrated. Factors such as trial design, patient selection, donor tissue preparation, and surgical techniques are important determinants of successful outcomes in clinical studies. Outcomes may therefore vary considerably depending on these factors, highlighting the complexity of achieving a consistent clinical benefit. ESCs also have a strong potential for teratoma formation and neural overgrowth [23,48,50], which may result in serious adverse effects, including graft-induced dyskinesias. In addition, ESCs can elicit a robust host immune response, causing additional complications [50,51,52]. Therefore, alternative cell sources have been developed by several research groups, as discussed below.

### 2.2. Induced Pluripotent Stem Cells

iPSCs are created by reprogramming adult cells to regain pluripotency and offer potential for many neurodegenerative diseases (for review, see Lee, et al. [53]). Much attention over the last decade has been focused on the therapeutic potential of iPSCs for several different neurologic conditions [53]. Human iPSCs [41] have an unlimited self-renewal capacity and differentiation potential, making them a promising cell type for cell replacement in chronic diseases. The process of genetic reprogramming, however, may introduce genomic and epigenetic alterations, including insertional mutagenesis, copy number variation, and epigenetic instability, which raise safety concerns and require careful quality control in clinical applications [54]. Human iPSCs have been used in both animal studies and, very recently, human clinical trials [34,45]. The first clinical trials using human iPSCs for cell replacement in PD patients were initiated in Japan in 2018 [38], followed by preclinical and clinical trials conducted by a group at Lund University in Sweden [30,55]. Schweitzer and collaborators recently reported a case study of a patient with PD who received an autograft of human iPSC-derived dopaminergic progenitors into the putamen [37]. At 18 to 24 months post-grafting, positron-emission tomography imaging suggested graft survival, and clinical observations indicated stabilization or improvement in PD symptoms [37]. In a small Phase I/II trial, Sawamoto, et al. [45] reported that iPSC-derived dopaminergic progenitor cells survived, produced DA, and did not form tumors. These results are promising and suggest that personalized medicine can help prevent graft rejection. Concerns remain, however, regarding the genetic stability of iPSCs and their potential for tumorigenicity due to the reprogramming process, which can introduce genetic mutations [56,57]. These risks highlight the need for continued research and refinement of iPSC technology to ensure its safety and efficacy in clinical applications. Despite these challenges, iPSC-based approaches remain a central focus of current translational research for cell replacement due to their capacity for scalable production of dopaminergic progenitors and their ongoing evaluation in early-phase clinical trials.

### 2.3. Clinical Translation of PSC-Derived Dopaminergic Progenitors

Recent advances in PSC differentiation protocols have enabled the clinical translation of PSC-derived dopaminergic neuron precursor therapies for PD, with multiple first-in-human trials now underway in North America, Europe, and Japan [39]. These trials employ ESCs or iPSC lines differentiated via floor plate-based developmental protocols and transplanted into the putamen using stereotactic techniques. While early-phase safety data are encouraging, long-term efficacy, optimal dosing, immune compatibility strategies, and genomic quality control considerations remain central challenges. For example, a recent Phase I trial of a human ESC-derived dopaminergic progenitor product (bemdaneprocel) demonstrated favorable safety and increased ^18^F-DOPA positron-emission tomography uptake, consistent with graft survival following bilateral putaminal transplantation in patients with PD [58].

Pluripotent stem cell-derived dopaminergic precursor therapies currently represent the most advanced regenerative strategy in clinical testing for PD [39]. The Kyoto Trial, initiated in 2018, enrolled seven patients in a Phase I/II design with safety as the primary endpoint [45]. Positron-emission tomography demonstrated graft survival and DA production, with four of six evaluable patients showing improvement in the motor examination component of the MDS-UPDRS (part III) during medication-off periods at 24 months [45]. Interpretation of these findings is limited, however, by the absence of a placebo control group, the small sample size, and the requirement for 15 months of tacrolimus-based immunosuppression. Hence, the long-term durability of clinical benefit remains uncertain [45]. Based on these early data, Japan’s Ministry of Health, Labor and Welfare granted conditional and time-limited marketing authorization in March 2026 for Amchepry (INN: raguneprocel; Sumitomo Pharma/RACTHERA) under its accelerated regenerative medicine framework, which permits conditional approval when safety is demonstrated and efficacy is considered predictive rather than definitively established [59]. Consequently, the therapeutic effectiveness of this approach must still be confirmed through post-marketing studies and longer-term follow-up [34]. These considerations underscore that although iPSC-derived dopaminergic progenitor therapy is scientifically promising, its current clinical evidence base remains limited and provisional [39,45]. In addition, practical challenges remain, including intracranial delivery requirements, the need for immunosuppression, genomic quality-control concerns during in vitro expansion, and graft heterogeneity [60,61,62]. These considerations underscore that, although PSC-derived dopaminergic progenitor therapy represents the leading regenerative strategy in clinical development, current evidence base remains early-stage and requires further validation in larger, controlled trials.

### 2.4. Mesenchymal Stromal/Stem Cells

MSCs offer practical advantages because they are relatively easy to obtain and expand in culture, exhibit favorable safety profiles, and raise minimal ethical concerns [63]. MSCs are collectible from bone marrow, umbilical cord, and adipose tissues from patients or donors [64]. When grafted MSCs are derived from the patient’s own tissue, no immunosuppressant treatments are required. MSCs from adult human bone marrow have been reported to differentiate into DA-like neurons under specific experimental conditions and neuroglia in culture and after transplantation into animal models, providing another potential source for cell replacement in humans with PD [35,46]. In initial experiments by Blandini, et al. [47], however, transplanted undifferentiated MSCs did not differentiate into a dopaminergic phenotype after grafting in rodents. Subsequent studies established protocols for modifying MSCs to induce a dopaminergic phenotype prior to transplantation [65]. MSCs induced to form neuronal cells have demonstrated promising results in experimental models of PD without forming tumors, in contrast to neural stem cells, iPSCs, and ESCs. The extent to which MSC-derived cells achieve a stable dopaminergic phenotype and functional integration in vivo, however, remains uncertain. They exhibit trophic and anti-inflammatory effects that support the repair of damaged tissues [63]. In PD, MSCs induced to form neuronal cells show potential to reduce motor symptoms and promote dopaminergic neuron survival [66]. Their therapeutic potential is enhanced by genetic modification, such as the introduction of the Notch1 intracellular domain and specific cytokines, which induce MSCs to produce dopaminergic neurons more efficiently [66]. Long-term studies are essential to ensure the safety and effectiveness of these genetically modified cells. MSCs face additional challenges for regenerative therapies, including immunosuppressive and pro-inflammatory activities; differences in therapeutic potential depending on the tissue source and donor characteristics; and risks such as undesired differentiation, tumor-promoting activity, and uncontrolled immune responses, the latter being highly dependent on the specific protocol used [67].

Extracellular vesicles (EVs), including exosomes, released from MSCs have also been investigated as a potential therapeutic strategy for PD (Table 1). In a mouse model of PD, Huang, et al. [68] showed that intranasal delivery of EVs derived from MSCs harvested from umbilical cord tissue protected against toxicity and reduced inflammation, suggesting that this approach may target several aspects of PD pathophysiology, most likely by providing trophic support to degenerating neurons. EVs produced by MSCs exert a strong neuroprotective effect, providing stable growth factors to support both dying neurons and developing cells in the graft [35]. Nevertheless, EV-based therapies do not directly replace lost dopaminergic neurons, highlighting the continued need to identify cell types capable of homing to the damaged region of the substantia nigra and restoring dopaminergic circuitry.

### 2.5. Limitations of Current Stem Cell Strategies for PD

Despite these advances, several obstacles remain in the development of stem cell-based therapies for PD. Placing the cells in the target region (i.e., caudate or putamen) requires a stereotaxic surgical approach via intracranial injection, an invasive procedure associated with surgical risk and logistical complexity [39]. While early safety reports are reassuring, the need for intracranial delivery remains a translational limitation. Although dopaminergic neurons degenerate in the substantia nigra, therapeutic grafts are typically placed in the striatum to restore dopamine signaling. Systemic or intranasal delivery approaches that permit targeted homing without the need for intracranial surgery or prolonged immunosuppression would represent a substantial translational advantage [68,69,70].

In addition to surgical considerations, most current stem cell-derived dopaminergic progenitor strategies rely on allogeneic transplantation and therefore require systemic immunosuppression to prevent graft rejection. Early-phase clinical trials using ESC- or iPSC-derived dopaminergic precursors incorporated prolonged immunosuppressive regimens, although the optimal duration and long-term consequences remain uncertain [39]. Systemic immunosuppression carries well-established risks, including infection, malignancy, and metabolic complications, and may be particularly concerning in an older population such as patients with PD [71]. Even with immunosuppression, immune-mediated graft responses have been reported in neural transplantation studies, highlighting the complexity of achieving durable engraftment in the human brain.

In addition, progenitor cells injected into the brain via intracranial stereotactic surgery may differentiate into other cell types after transplantation, thereby reducing efficacy and potentially causing complications, including teratomas and/or other side effects of the grafting procedure [44,72,73]. Tiklova, et al. [72] demonstrated that stem cell grafts can differentiate into neurons, astrocytes, and perivascular cells within stem cell-derived grafts; if not carefully controlled, this cellular heterogeneity may be problematic. Tumor formation following the transplantation of ESCs or iPSCs into the brain hampered earlier attempts to use stem cells for cell replacement [48,49,74]. In addition to concerns regarding residual undifferentiated cells, PSCs may accumulate genomic aberrations during in vitro expansion, including structural variants that are not always detectable by conventional karyotyping. Recent analyses emphasize the need for advanced genomic quality-control strategies to ensure long-term safety of PSC-derived grafts intended for durable CNS engraftment [39]. Moreover, uncontrolled differentiation into various cell types could mean that only a minority of the cells in a graft represent the cell phenotype needed for replacement. Careful selection of stem cell types, differentiation protocols, and thorough preclinical testing are critical to avoid this potential complication [34]. Given these challenges—including invasive delivery, cellular heterogeneity, tumorigenic risk, and the need for systemic immunosuppression—alternative cell sources are needed.

Multilineage-differentiating Stress-Enduring cells (Muse cells) have emerged as an alternative and mechanistically distinct regenerative platform for PD therapy. Muse cells represent a unique pluripotent-like subpopulation of MSCs that can differentiate into dopaminergic neurons without genetic manipulation, thereby reducing the risk of tumor formation [75,76]. Muse cells have the inherent ability to home to sites of tissue damage following intravenous administration and integrate into the host environment, making them an attractive candidate for further research in PD cell replacement therapy. This rationale is strengthened by evidence that Muse cells have demonstrated safety and therapeutic benefit in controlled, multi-dose clinical trials for other conditions with comparable neurodegeneration and have been administered intravenously without HLA matching or immunosuppression [60,61,62].

Muse cells, with their unique characteristics, represent a biologically distinct alternative strategy that may overcome many of the limitations associated with current stem cell therapies, paving the way for more effective, disease-modifying treatments. In this review, we present a new cell source that circumvents previous potential obstacles in current cell replacement strategies. It is important to distinguish between true dopaminergic cell replacement and broader regenerative or supportive effects. Improvements observed in preclinical models may reflect neuroprotection, anti-inflammatory activity, or trophic support rather than restoration of dopaminergic circuitry. Demonstration of true cell replacement requires evidence of functional integration, synaptic connectivity, and sustained dopaminergic signaling within the nigrostriatal pathway. In the following sections, we examine the biologic properties of Muse cells and evaluate their potential as a regeneration platform for PD.

## 3. Biologic Basis of Muse Cells for Regenerative Therapy

**Figure 1:**
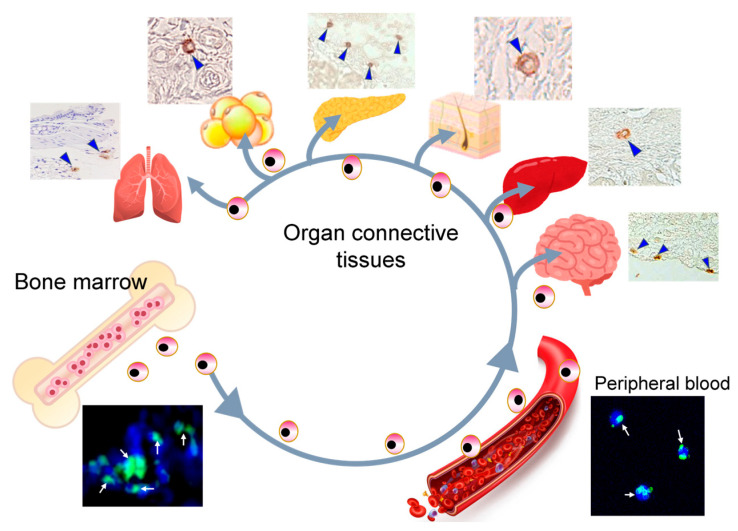
Tissue sources of Muse cells. Distribution of endogenous Muse cells. Stage specific-embryonic antigen-3–positive Muse cells in the bone marrow and peripheral blood are labeled with green fluorescence, and the Muse cells in the connective tissue of each organ are colored brown.

Muse cells are endogenous reparative stem cells with pluripotent-like properties and macrophage-like behavior. They are present in bone marrow (0.01–0.03% of the mononuclear fraction), peripheral blood (0.01–0.2% of the mononuclear fraction), and connective tissues of multiple organs as stage specific-embryonic antigen-3–positive cells (Figure 1) [77,78]. They are also detectable as a small subpopulation within cultured MSCs and fibroblasts [77,79]. Unlike ESCs and iPSCs, Muse cells do not form teratomas in immunodeficient mice and display proliferation rates comparable to fibroblasts, enabling clinical-scale expansion without evidence of tumorigenicity in preclinical models [79,80,81,82].

Systemically administered Muse cells selectively home to damaged tissue via the sphingosine-1-phosphate (S1P)-S1P receptor 2 (S1PR2) axis, where they differentiate into tissue-specific cells corresponding to the site of injury and integrate into the host tissue in preclinical and early studies, including reports without HLA matching or long-term immunosuppression [60,61,62]. The long-term durability and generalizability of these immune-related observations, however, remain incompletely characterized. This combination of targeted homing, site-appropriate differentiation, and immune tolerance mechanistically distinguishes Muse cells from other stem cell populations.

### 3.1. Core Characteristics

Muse cells express pluripotency-associated genes, including Nanog, Oct3/4, Sox2, Rex1, and PAR4, at moderate levels compared with ESCs and iPSCs, and demonstrate triploblastic-lineage differentiation at the single-cell level with self-renewal capacity [77,79]. Unlike conventional PSCs, however, Muse cells couple stemness with innate reparative behavior.

They selectively migrate toward S1P gradients released by injured cells and recognize phosphatidyl serine on apoptotic membranes, as shown in animal models [83,84]. This injury-responsive sensing links migration to site-specific differentiation and underlies their endogenous reparative function [85].

### 3.2. Phagocytosis-Driven Differentiation

**Figure 2:**
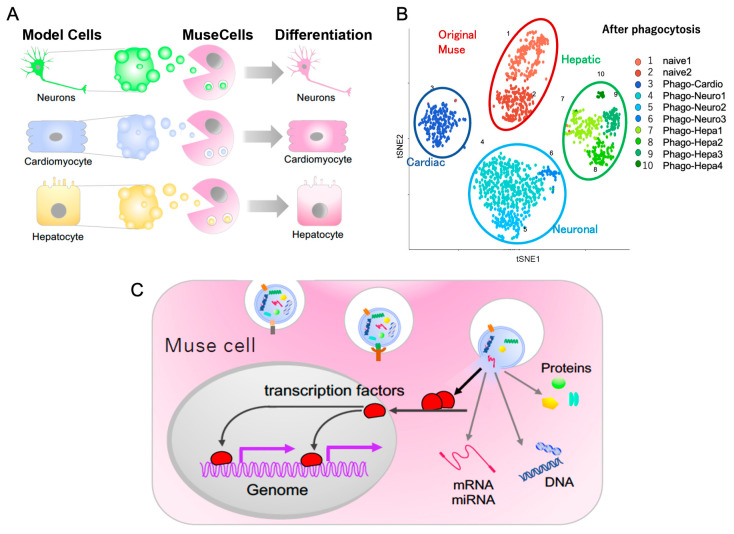
Muse cells phagocytose damaged cells to differentiate into triploblastic-lineage cells [84]. (**A**) Muse cells home to damaged cells, phagocytose the cells, and differentiate into lineage-specific cells. (**B**) Single-cell RNA sequencing of human Muse cells after phagocytosing apoptotic cell fragments of mouse hepatic (hepatic), mouse cardiac (cardiac), and rat neural (neuronal) cells (within 7 days). (**C**) The mechanism by which Muse cells recycle signals from the phagocytosed damaged/apoptotic cells necessary for differentiation, such as transcription factors. From Minatoguchi, et al. [60].

After homing to injured cells, Muse cells initiate lineage specification through phagocytosis of apoptotic or damaged cell fragments. This process is mediated by receptors, including CD36, RAGE, integrin β3, and CD91/LRP-1 [84]. Transcription factors derived from engulfed cells are internalized and translocate to the Muse cell nucleus, where they activate lineage-appropriate gene expression programs that drive differentiation (Figure 2A,C) [60,84].

Single-cell RNA sequencing demonstrates rapid acquisition of tissue-specific transcriptional profiles within days following phagocytosis, including neural lineage signatures after exposure to neural debris (Figure 2B) [84]. Inhibition of phagocytic signaling markedly attenuates this differentiation, indicating that phagocytosis is not merely supportive but instructive for fate determination. Consistent with these in vitro findings, Wakao, et al. [84] also provided supportive in vivo evidence in a mouse focal stroke model: transplanted Muse cells contained host-derived brain tissue fragments within LAMP-1-positive phagosomes, and cells that had phagocytosed these fragments expressed a neuronal differentiation reporter, whereas suppression of phagocytosis reduced subsequent neuronal differentiation. These findings suggest that phagocytosis may help guide environment-specific differentiation of Muse cells in vivo as well as in vitro, although translational findings in patients with neurologic conditions are yet to confirm these initial findings in translational or clinical studies because this field is still young.

### 3.3. Injury-Directed Migration and Relevance to PD

**Figure 3:**
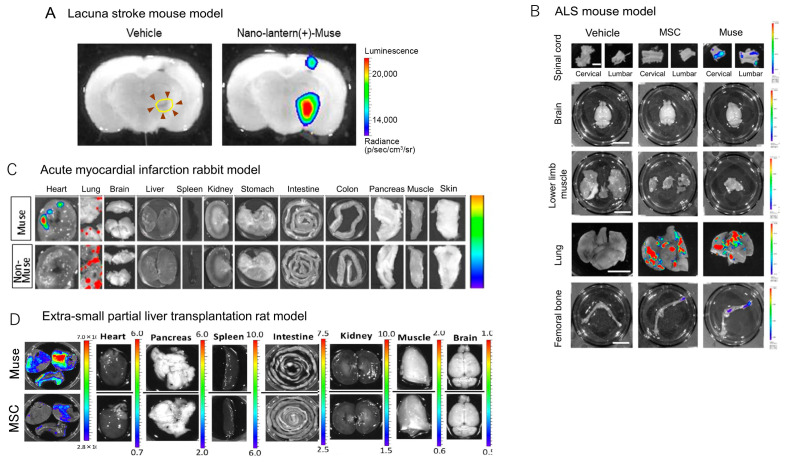
Biodistribution of intravenously injected Muse cells in disease models. (**A**) Mouse lacunar stroke model that received Nano-lantern-labeled human bone marrow-Muse cells. Homing of Nano-lantern–labeled human Muse cells to the infarct region 1 day after infarct [69]. Figure reproduced from Abe, et al. [69]. (**B**) Mouse G93A-transgenic ALS model that received Nano-lantern-labeled human bone marrow-Muse cells/MSCs 7 days after. In addition to the lung, homing of Muse cells was detected in the cervical and lumbar spinal cord, while that of MSCs was under the detection limit [86]. Figure reproduced from Yamashita, et al. [86]. (**C**) Rabbit acute myocardial infarction model that received Nano-lantern-labeled human bone marrow-Muse cells/non-Muse MSCs at 3 days. Muse cells preferentially homed to the post-infarct heart, while non-Muse MSCs demonstrated limited homing to the injured tissue and were largely retained in the lung in comparative studies [83]. Figure reproduced from Dezawa [85] (**D**) Extra-small partial liver transplantation rat model that received Akaluc-labeled human bone marrow-Muse cells/MSCs at 3 days. Muse cells were actively detected in the liver compared to MSCs [87]. Figure reproduced from Shono, et al. [87].

Muse cell homing is driven by S1P-S1PR2 signaling [83]. In models of stroke and amyotrophic lateral sclerosis (ALS), intravenously administered Muse cells selectively accumulate in regions of injury (Figure 3A,B), whereas non-Muse MSCs exhibit minimal CNS integration [69,86]. In the stroke mouse model, Muse cells accumulated in the ischemic region 1 day after intravenous injection (Figure 3A). In the ALS mouse model, Muse cells were detected in the cervical and lumbar spinal cord at 7 days after intravenous injection, whereas MSCs were under the detection limit in the same regions (Figure 3B). A similar behavioral difference between Muse cells and MSCs/non-Muse MSCs after intravenous injection was demonstrated in acute myocardial infarction (Figure 3C) and extra-small partial liver transplantation models (Figure 3D), in which Muse cells actively accumulated in the post-infarct heart tissue and transplanted liver, respectively, at 3 days after intravenous injections, while non-Muse MSCs and MSCs were under the detection limit on the same day [87]. These basic experiments suggest that a major difference between the integration of Muse cells and MSCs/non-Muse MSCs into injured brain regions appears to be related specifically to the homing ability of Muse cells, although these findings require confirmation across multiple models and in human clinical trials. The therapeutic window appears broader in the CNS than in peripheral tissues, with homing observed during subacute and chronic phases of injury [69].

Recent evidence extends this mechanism to PD models. In a transgenic A53T mouse model of PD [88], intranasally administered human Muse cells were detected in degenerating brain regions and demonstrated enhanced transmigration across an in vitro blood–brain barrier under inflammatory conditions via S1PR2–Rho signaling [70]. These mice overexpress the human A53T mutant α-synuclein and develop severe movement dysfunction, paralysis, neuronal degeneration and death, and synucleinopathy in cortical and subcortical brain regions [88]. Inhibition of S1PR2 reduced brain homing, confirming pathway specificity, at least in this PD mouse model. These findings provide PD-relevant evidence that systematically administered Muse cells can access neurodegenerative brain regions through injury-directed migration mechanisms, although confirmation across additional PD models is required. The PD model used in these preliminary cell replacement experiments may not fully capture the pathologic diversity and heterogeneous symptom progression characteristic of human PD.

### 3.4. Evidence for Direct Contribution of Muse Cells to Functional Recovery

While paracrine and immunomodulatory effects contribute to tissue repair, several studies indicate that engraftment and differentiation of Muse cells also play a role in functional recovery. In a rat spinal cord injury model, intravenously administered human Muse cells preferentially homed to the spinal cord and differentiated into neural lineage cells, with a majority expressing neuronal markers such as MAP-2 [89]. Functional improvement was observed over an 8-week period. Selective ablation of engrafted human cells using diphtheria toxin led to a significant decline in motor function, indicating that Muse-derived cells were required to sustain the therapeutic effect [89].

In a rabbit model of acute myocardial infarction, Muse cells preferentially homed to injured myocardium via the S1P–S1PR2 axis, engrafted, and expressed cardiac and vascular lineage markers, including cardiac troponin-I and connexin-43, while non-Muse cells resulted in very little engraftment in the post-infarct cardiac tissue when S1PR2 was inhibited [83]. Intravenous administration of Muse cells whose cardiomyocyte differentiation was inhibited by GATA-4 siRNA, as well as elimination of Muse cells using suicide genes after engraftment and differentiation in the post-infarct region, significantly reduced the cardiac repair effects [83].

Together, these findings indicate that engraftment and lineage-specific differentiation of Muse cells contribute to functional recovery, alongside bystander mechanisms.

### 3.5. Neural and Dopaminergic Differentiation

**Figure 4:**
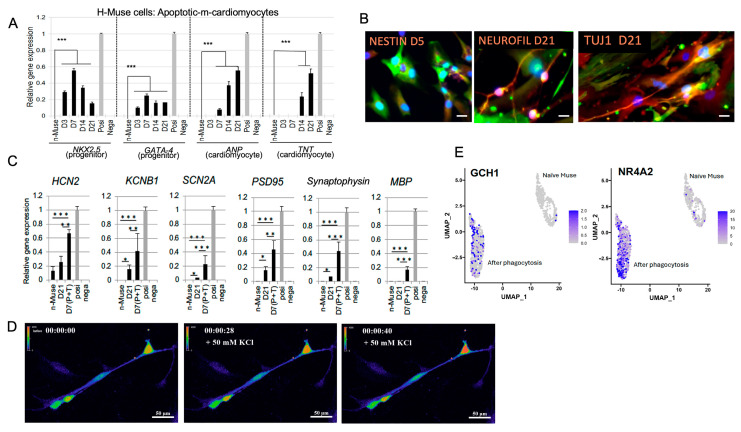
Muse cells phagocytose neuronal cell fragments and undergo neural lineage differentiation [84]. (**A**,**C**) Quantitative polymerase chain reaction of human neural markers in naive Muse cells (n-Muse) and Muse cells after incubating with apoptotic fragments derived from primary cultured rat hippocampus (r-neural cells) from D3 to D21. After incubation with neuronal cell fragments for 3 days, human-Muse cells were further co-cultured with damaged rat brain tissue slices for 7 days (D7 [P + T]) (mean ± SEM). (**B**) Immunocytochemical staining showing neuronal marker expression in human Muse cells after phagocytosing apoptotic rat hippocampal fragments. (**D**) Intracellular calcium dynamics in green fluorescent protein-based Ca calmodulin probe (GCaMP)-h-Muse cells after biochemical depolarization with 70 mM KCl. (**E**) Single-cell RNA sequencing of human Muse cells 7 days after phagocytosing apoptotic rat neural cell fragments revealed neuronal lineage-associated gene expression, including genes linked to dopaminergic neuronal function (e.g., GCH1 and NR4A2). * *p* < 0.05, ** *p* < 0.01, *** *p* < 0.001. Panels (**A**–**D**) are reproduced from Wakao, et al. [84]. Panel (**E**) shows the results of a reanalysis of single-cell RNA-sequencing data from Wakao, et al. [84], which are deposited in ArrayExpress under accession ID E-MTAB-16736.

Muse cells demonstrate the capacity for neural differentiation both in vitro and in vivo. Following exposure to apoptotic neural cell fragments, human Muse cells upregulate neural-lineage–associated markers, including FOXP2, PAX6, MATH1, NEUN; express synaptic markers such as synaptophysin and PSD95; and exhibit depolarization-induced calcium influx consistent with neuronal maturation (Figure 4A–D) [84]. Importantly, differentiated Muse cells expressed markers related to midbrain dopaminergic neurons, such as GCH1 (involved in DA synthesis, expressed in the nerve terminals of dopaminergic neurons in the substantia nigra pars compacta) and NR4A2 (plays a key role in the maintenance of the dopaminergic system of the brain), suggesting the potential of Muse cells to acquire dopaminergic neuronal phenotypes (Figure 4E).

Directed differentiation protocols further support this neurogenic capacity of Muse cells under controlled experimental conditions. Under defined culture conditions, Muse cell-derived neural progenitors can be specified to become dopaminergic neurons expressing tyrosine hydroxylase and the DA transporter, along with additional markers of neuronal maturation, without genetic reprogramming [90]. This distinction is relevant when considering genomic stability concerns associated with iPSC–based approaches.

In vivo evidence supports the feasibility of neural differentiation within injured brain tissue. In models of CNS injury, systemically administered Muse cells home to sites of damage, integrate into host tissue, and express neuronal markers [69,86]. Evidence from PD models further supports the capacity of Muse cells to undergo dopaminergic differentiation in vivo. In a transgenic A53T mouse model of PD, intranasally administered human Muse cells homed to neurodegenerating brain regions and expressed tyrosine hydroxylase in vivo, indicating differentiation toward a dopaminergic phenotype within the Parkinsonian microenvironment [70]. While these studies demonstrate dopaminergic marker expression, robust lineage tracing, functional validation, long-term reconstruction of the nigrostriatal pathway, and sustained behavioral recovery in PD models remain to be established.

Together, these findings indicate that Muse cells possess both intrinsic and injury-responsive mechanisms that support neural and dopaminergic differentiation in experimental settings. Additional preclinical validation is required, however, to determine the extent of functional integration and sustained dopaminergic activity in PD. Accordingly, current evidence supports further investigation of Muse cells as an experimental candidate for dopaminergic cell replacement. These studies should follow the same experimental protocols and rigorous testing used for other stem cell sources that are now in clinical trials for PD patients (see, e.g., Christiansen and Kirkeby [34]).

### 3.6. Pleiotropic and Microenvironment-Modulating Effects

Beyond their ability to replace damaged cells, Muse cells exhibit bystander effects, such as anti-inflammatory, antifibrotic, anti-apoptotic, neovascularization, and tissue-protective effects, which may be relevant to PD. These effects are mediated in part through the secretion of immunomodulatory and trophic factors, including interleukin-10, transforming growth factor-β, prostaglandin E2, vascular endothelial growth factor (VEGF), platelet-derived growth factor, fibroblast growth factor, granulocyte colony-stimulating factor, and hepatocyte growth factor [91,92,93,94]. Muse cells also produce matrix metalloproteases-1, -2, and -9, which suppress fibrosis [83]. Overall, these properties suggest that Muse cells may contribute to tissue repair through multiple complementary mechanisms, including rapid and selective homing to sites of injury, differentiation into tissue-specific cells, and microenvironment-modulating bystander effects.

Recent studies demonstrated that MSCs produce and secrete growth factors, trophic factors, and cytokines when grafted into PD mouse models and patients, and that the main therapeutic mechanism of MSC engraftment is mediated by these factors, creating a supportive environment for surviving neurons, rather than replacing dopaminergic SN neurons [95]. Muse cells also produce and secrete epidermal growth factor (EGF) and insulin-like growth factor, known to protect dopaminergic neurons [92]. In addition, the production of VEGF and angiopoietin-1 may contribute to vascular stability of blood flow and tissue perfusion [92]. In the rat lung injury model, intravenous administration of human-bone marrow-derived Muse cells increased expression of several anti-apoptosis factors, including Bcl-2 and Akt, suggesting potential cytoprotective effects that may be relevant in neurodegenerative settings [92]. It is therefore possible that Muse cells provide both a nurturing anti-apoptotic environment and direct cell replacement when administered in mouse models or patients with PD, although this has yet to be explored in translational experiments.

### 3.7. Immunologic Profile

Allogeneic Muse cells have been administered intravenously in preclinical and early clinical studies, including reports without HLA matching or prolonged immunosuppression [61,62,96,97]. Proposed mechanisms include HLA-G expression and secretion of immunomodulatory factors such as transforming growth factor-β, prostaglandin E2, indoleamine 2, 3-dioxygenase, and nitric oxide [83,91,92]. Xenogeneic human Muse cells administered intravenously into a rat neonatal hypoxic–ischemic encephalopathy model have been reported to evade overt immune rejection without immunosuppression under specific experimental conditions [98].

Most allogeneic cells without HLA matching or immunosuppression are quickly rejected by the host’s immune system [99]. The ability to administer HLA-mismatched allogeneic Muse cells without immunosuppression may be relevant to clinical applications, given that immunosuppressive therapy increases the risk of infection [100]. This immune tolerance may be particularly advantageous in diseases like PD, where immune-mediated inflammation contributes to pathology, and it may distinguish Muse cells from iPSC-derived allogeneic grafts, which typically require immunosuppression, although direct comparative studies remain limited. On the other hand, as Muse cells can be produced from the patient’s own bone marrow or adipose cells [101], autologous transplantation may ultimately be feasible, potentially eliminating the risk of immune rejection altogether.

### 3.8. Non-Tumorigenicity

Muse cells exhibit enhanced DNA repair capacity and low tumorigenicity, distinguishing them from other stem cell types, such as MSCs [102,103]. They demonstrate increased resistance to stressors such as ultraviolet light and H_2_O_2_, exhibiting reduced apoptosis and cellular senescence. This resilience is associated with elevated expression of DNA repair proteins, including ataxia-telangiectasia mutated kinase and γ-H2AX, as well as increased activity of enzymes involved in non-homologous end-joining repair pathways [103]. Despite their pluripotency, Muse cells display telomerase activity comparable to that of somatic cells such as fibroblasts [79], and, in contrast to ESCs and iPSCs, they do not form teratomas when transplanted into immunodeficient mice [79,80,81,82]. Additionally, Muse cells lack expression of the pluripotency-associated protein LIN28 and instead have high levels of the tumor-suppressor microRNA let-7 [104,105,106]. Let-7 inhibits the PI3K-AKT pathway, modulating pluripotency-associated transcription factors including KLF4, Oct3/4, SOX2, and NANOG, while limiting proliferation and glycolysis [106]. In Muse cells, self-renewal and senescence suppression are primarily regulated by the MEK/ERK pathway rather than the PI3K-AKT axis. This regulatory configuration, characterized by let-7–mediated control of pluripotency gene expression in the absence of oncogenic LIN28, appears to permit pluripotency-like functionality without activating proliferative oncogenic pathways and may contribute to the low tumorigenic profile observed in preclinical models. Although these features support a favorable safety profile in preclinical models, long-term clinical safety data remain limited.

## 4. Muse Cells in Preclinical Disease Models

### 4.1. Parkinson’s Disease

As noted above, preclinical evidence for Muse cells in PD is currently limited to a single recent study by Lu, et al. [70] using an A53T transgenic mouse model. In that report, human Muse cells were administered intranasally and were subsequently detected in the brain, including the cerebral cortex and substantia nigra, whereas non-Muse MSC cells were largely absent from these regions and appeared to accumulate preferentially in the lungs. Muse cell treatment was associated with a higher number of tyrosine hydroxylase-positive and NeuN-positive cells in the substantia nigra, reduced neuronal damage on histologic assessment, lower tumor necrosis factor-α and S1P expression, and increased expression of the neurotrophic factors BDNF and GDNF relative to untreated and non-Muse cell-treated A53T mice. Behavioral improvement was also reported, with shorter climbing and turning times in the pole test and longer suspension times in the wire-hang test after Muse cell administration. Mechanistically, the authors proposed that Muse cells may cross a compromised blood–brain barrier through an S1P-S1PR2-dependent pathway, based on in vitro blood–brain barrier experiments, higher S1PR2 expression in Muse than non-Muse cells, and attenuation of migration and in vivo benefit following S1PR2 antagonism [70]. Although these findings support the potential neuroprotective effects of Muse cells in PD, the evidence remains preliminary and will require replication in additional synuclein-based and toxin-based models, as well as longer-term studies to address durability, cell fate, and safety. Importantly, the observed improvements may reflect a combination of neuroprotective, anti-inflammatory, and trophic effects rather than definitive evidence of functional dopaminergic cell replacement or reconstruction of the nigrostriatal pathway.

### 4.2. Acute, Subacute, and Chronic Rodent Stroke Models

**Figure 5:**
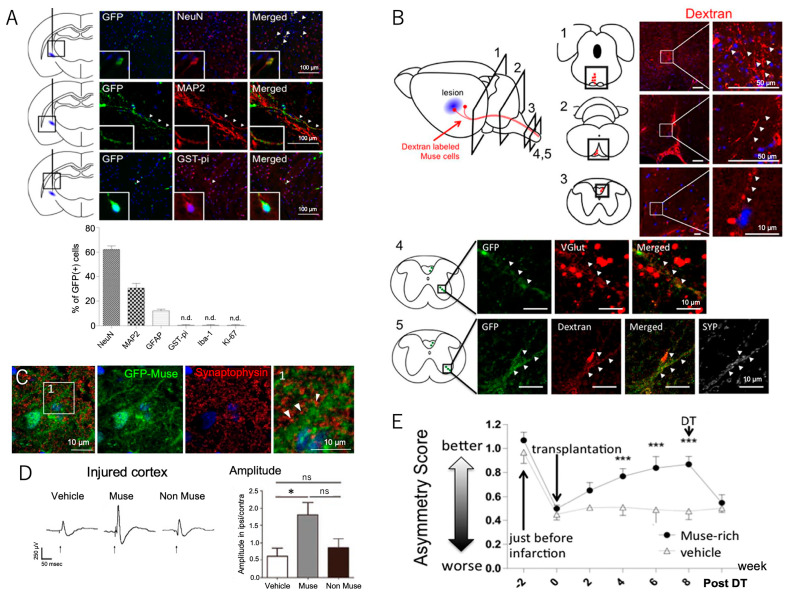
Rodent stroke models that received human Muse cell treatment [69,107,108]. (**A**,**B**) Human Muse cells injected perilesionally 2 weeks after inducing a lacunar infarct in mice [108]. (**A**) Differentiation of green fluorescent protein (GFP)-Muse cells into neuronal (NeuN, MAP2)-, oligodendrocyte (GST-pi)-, astroglial (GFAP)-, microglial (Iba-1)-, and proliferative cell (ki-67)-markers. n.d.: not detected (**B**) Dextran-labeled axons (red, arrowheads) were detected at level 1: midbrain and level 2: medulla on the ipsilateral side and in the cervical spinal cord at levels 3 to 5 on the contralateral side 8 weeks after. Levels 4 and 5 show the area of the anterior horn in the spinal cord where pyramidal tract axons form synapses with motor neurons. GFP+ neurite positive for VGlut, a glutamatergic neuron marker (level 4). Dextran (red)–labeled GFP (green)–positive Muse cells were positive for synaptophysin (white) in the upper cervical spinal cord (level 5) [108]. Scale bars, 100 μm (**A**), 50 μm ((**B**): levels 1 and 2) and 10 μm ((**B**): levels 3–5). (**C**,**D**) Rat middle cerebral artery occlusion model received a stereotaxic injection of human Muse cells 2 days after occlusion and subjected to histologic and electrophysiologic analyses at day 84 post-occlusion [107]. (**C**) In the ipsilateral sensory cortex, synaptophysin (red) was detected adjacent to dendrite-like structures of GFP-labeled Muse cells (green) (arrowheads). Scale bar = 10 μm. 1 is an enlarged image of the boxed area in the left panel. (**D**) Somatosensory evoked potentials in the vehicle, Muse, and non-Muse groups in the ipsilateral sensory cortex. The graph shows the ratio of the ipsilateral somatosensory-evoked potential amplitude to that on the contralateral side. ns: not significant, *: *p* < 0.05. (**E**) Loss of function experiment by intraperitoneal injection of diphtheria toxin (DT) [60,69]. ***: *p* < 0.001. Figures were reproduced from Uchida, et al. [107], Uchida, et al. [108], and Minatoguchi, et al. [60].

Muse cells demonstrate significant therapeutic potential in preclinical stroke models. In a rat lacunar stroke model, intravenously injected Muse cells selectively migrated to the post-infarct area by day 1 and expressed neuronal and oligodendrocyte markers (Figure 3 and Figure 5A) [69,107]. Human Muse cells expressed early neural markers Mash1 and NeuroD within ~3 days and maturity markers MAP2 and NeuN, as well as the glial cell marker GST-pi, within 7–14 days (Figure 5A) [107,108]. The human Muse cell-derived neuronal cells were incorporated into the pyramidal tract, including the pyramidal decussation, as demonstrated by retrograde and anterograde tracing (Figure 5B) [107,108]. Similarly, they were incorporated into the sensory tracts, as evidenced by the recovery of somatosensory-evoked potentials and the formation of synapses with host neuronal cells (Figure 5C,D). The Muse cell-treated group exhibited significant therapeutic effects in motor (modified neurologic severity score, rotarod, corner turn, and cylinder tests) and sensory function scores compared with vehicle and MSC groups within 3 months [107,108].

When integrated Muse cells were eliminated by a loss-of-function experiment at 8 weeks, the time at which functional recovery was stable in the cylinder test, compared with the vehicle group, active recovery was abolished and dropped sharply to the vehicle group level, suggesting that integrated Muse cells mediated the behavioral outcome (Figure 5E) [69,108].

A similar trend was observed following intravenous injection of clinical-grade human Muse cells into a mouse lacunar stroke model at both subacute (day 9) and chronic (day 30) phases [69]. In both cases, human Muse cells expressed neuronal and glial cell markers in the peri-infarct region. The extent of functional recovery was dose-dependent and was observed even when the human Muse cell injection was administered on day 30. Persistent therapeutic effects were confirmed for up to 22 weeks with no striking adverse effects [69]. No tumor formation was observed in the brain, lung, kidney, liver, or spleen, and Muse cells did not disseminate to other tissues for up to 10 months [69,108].

Together, these results suggest that Muse cells may exert therapeutic effects by replacing neural cells, contributing to the reconstruction of neuronal circuits. These findings are derived from stroke models, however, and their direct relevance to PD remains uncertain—particularly given differences in disease mechanisms, patterns of neurodegeneration, and requirements for dopaminergic circuit reconstruction.

### 4.3. Chronic Model: Mouse ALS Model

The therapeutic potential of human Muse cells and MSCs has also been evaluated in a chronic neurodegenerative disease model of ALS using SOD1-G93A transgenic mice expressing the G93A mutant form of human SOD1, which exhibit a phenotype similar to that of ALS patients [109]. Although PD and ALS are distinct clinical conditions affecting different brain and spinal cord regions, they share fundamental pathologic processes [110]. ALS and PD are classified as proteinopathies and neurodegenerative diseases that are characterized by protein misfolding, neuroinflammation, and cellular stress causing mitochondrial dysfunction [110]. Intravenous injection was more efficient than intrathecal injection in delivering Muse cells to the cervical and lumbar spinal cord, the therapeutic target for ALS, whereas MSCs were under the detection limit in the spinal cord post-injection [86]. The therapeutic effects were compared among mice intravenously administered human Muse cells, MSCs, or vehicle based on behavioral test scores, motor neuron survival, and myofiber atrophy. Compared with the MSC and vehicle groups, the Muse cell group showed statistically significant therapeutic effects, including improvements in behavioral test scores, enhanced motor neuron survival in the lumbar spinal cord, reduced myofiber atrophy, and neuromuscular junctions in the tibialis anterior muscle at various time points for at least 154 days.

These findings suggest that Muse cells, which can reach the target spinal cord by intravenous injection and deliver therapeutic effects, are a potential treatment option for other chronic neurologic diseases with similar disease mechanisms as ALS (see Section 5.2). Although providing evidence for a potential clinical application, these findings do not directly establish efficacy in PD and should be interpreted as supportive but indirect evidence of therapeutic potential.

## 5. Clinical Trials

Muse cell therapy has been reported in preclinical and early clinical studies without requiring HLA-matching, immunosuppressive therapy, surgical delivery, or genetic manipulation before transplantation. Following intravenous administration, Muse cells home to sites of tissue damage, phagocytose apoptotic cell debris, and differentiate into the corresponding cell type in vivo with few errors [60].

In addition to these characteristics, Muse cells exert pleiotropic effects, including anti-fibrotic, anti-inflammatory, and anti-apoptotic activities, as well as tissue and vascular protection. Because Muse cells, including allogeneic Muse cells, are reported to survive and integrate into host tissues in preclinical and early clinical studies, their effects may be sustained. Their stress tolerance and low risk of tumorigenesis further support their potential for treating diseases involving tissue destruction. Clinical trials involving intravenous administration of donor-derived Muse cells without HLA matching or immunosuppressive therapy have been conducted for subacute ischemic stroke, ALS, cervical spinal cord injury, neonatal hypoxic–ischemic encephalopathy, acute myocardial infarction, and epidermolysis bullosa [60]. These trials demonstrate acceptable short-term safety profiles and preliminary signals of potential therapeutic benefit but should be interpreted cautiously given the limited sample size and early-phase nature of the studies [60,111,112]. This section focuses on the outcomes of clinical trials for stroke and ALS, the neurodegenerative conditions for which Muse cells have been most extensively evaluated to date.

### 5.1. Subacute Stroke

**Figure 6:**
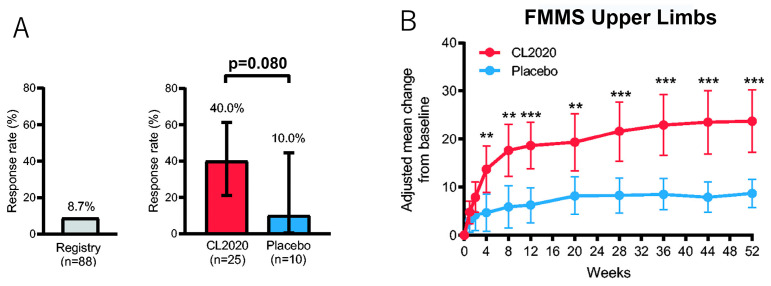
Clinical trial in subacute stroke. (**A**) Responder analysis of patients with modified Rankin Scale (mRS) scores of 0–2 at 12 weeks, demonstrating mean (95% confidence interval) response rates in the CL2020 (40.0% [21.1, 61.3]) and placebo (10.0% [0.3, 44.5]) groups; the threshold response rate of 8.7% based on registry data. The lower limit of the 95% CI in the CL2020 group was higher than the 8.7% seen in the registry data. The difference between the CL2020 and placebo groups was analyzed by Fisher’s exact test (mid-*p* value). (**B**) Adjusted mean (95% confidence interval) change from baseline in the Fugl-Meyer Motor Scale upper limb score in the CL2020 (*n* = 19–25) and placebo (*n* = 8–10) groups. **: *p* = 0.01, ***: *p* = 0.001. Figures were reproduced from Niizuma et al. [61].

A double-blind, placebo-controlled randomized clinical trial enrolled ischemic stroke patients with a modified Rankin Scale (mRS) score ≥3 [61]. Patients were administered a single intravenous dose of either ~1.5 × 10^7^ clinical-grade donor-Muse cells (CL2020; *n* = 25) or placebo (*n* = 10) without immunosuppression 14–28 days after onset. Safety (primary endpoint at 12 weeks) and efficacy (mRS and other stroke-specific measures) were evaluated for up to 52 weeks. The key efficacy endpoint was the response rate (percentage of patients with mRS score ≤2 at 12 weeks). At 12 weeks, adverse reactions were experienced in 28% of the Muse cell group (including one Grade 4 status epilepticus) and 10% of the placebo group. Although the study was conducted without immunosuppression, no serious adverse reactions attributable to rejection were observed. The response rate was 40.0% (95% confidence interval, 21.1, 61.3) in the Muse cell group and 10.0% (0.3, 44.5) in the placebo group (Figure 6A) [61].

Significant improvements in the Fugl-Meyer Motor Scale upper limb and total scores were observed in as little as 4 weeks and sustained through 52 weeks, with corresponding improvements in mRS scores to 0 or 1 [61]. Such early recovery of upper limb function is uncommon, and previous studies indicate that upper limb function is essential for many activities of daily living (e.g., dressing and eating) and is therefore highly correlated with the recovery of patient independence (Figure 6B) [113].

Loss-of-function experiments in a mouse lacunar stroke model further demonstrated that eliminating Muse cells that had integrated as neural cells rapidly abolished the observed functional improvement [69,108]. In humans as well, sustained functional improvement may depend on the persistence of engrafted Muse cells, although this relationship has not yet been directly established. Sustained improvement may reflect continued activity of engrafted Muse cells or indirect neuroprotective effects on the host environment.

Overall, intravenous administration of donor Muse cells has demonstrated acceptable short-term safety and preliminary evidence of therapeutic activity in early-phase studies conducted without HLA matching or immunosuppressants, although larger, controlled studies are required to confirm efficacy [60]. Although no serious adverse reactions attributable to immune rejection were observed, longer-term immune responses and durability of engraftment remain incompletely characterized.

### 5.2. ALS

Unlike in the clinical trials for ischemic stroke, in which tissue damage occurs suddenly, clinical-grade Muse cells were administered multiple times to patients with ALS, in which motor neuron degeneration progresses slowly and continuously. To confirm the safety of multiple intravenous injections of Muse cells, five patients with sporadic ALS with the limb-onset clinical form were enrolled in an open-label, non-randomized, single-arm, non-controlled clinical trial [62]. Patients received monthly intravenous injections of ~1.5 × 10^7^ clinical-grade Muse cells for 6 months. The primary endpoints were safety and tolerability, and the secondary endpoint was the rate of change in the Revised Amyotrophic Lateral Sclerosis Functional Rating Scale (ALSFRS-R) score for up to 12 months.

Repeated administration of donor-derived Muse cells without HLA-matching or immunosuppressive therapy was well tolerated, with no reported cases of pulmonary embolism, anaphylactic shock, or other serious adverse events. The most common adverse events were headaches and fatigue. ALSFRS-R scores remained stable in four patients during the observation period. The remaining patient exhibited a tendency toward slower disease progression for up to 10 months after initiating Muse cell treatment. These results suggest potential therapeutic effects of Muse cell treatment in ALS, although conclusions are limited by the small sample size and study design.

Overall, these findings indicate a favorable safety and tolerability profile for Muse cell therapy and suggest a possible effect on disease progression in ALS. The small sample size and absence of a control group limit interpretation, and larger, controlled studies are necessary to fully assess clinical efficacy. Alzheimer’s disease (AD) shares significant overlapping pathologic and clinical features with ALS and PD, including misfolding and aggregation of toxic peptides leading to massive and progressive neuronal loss [114]. Although preliminary studies investigating stem cell replacement therapies, including Muse cells, in AD, are in their infancy, they show considerable promise for the future of cell replacement strategies in this most common neurologic disease.

## 6. Similarities and Differences Between Muse Cells and MSCs

Muse cells, first discovered as a stress-tolerant subpopulation of bone marrow-MSCs corresponding to ~1% to several percent of the total MSC population, have several characteristics in common with MSCs, such as the expression of mesenchymal surface markers (CD29, CD44, CD90, CD73, and CD105), and pleiotropic effects such as cytokine production, activation of endogenous tissue stem cells/progenitors, vascular protection, neovascularization, immune modulation, and inflammation-suppression, activities that synergistically contribute to creating a microenvironment supportive of tissue repair [77,115,116,117].

On the other hand, Muse cells exhibit unique characteristics that may differ from those observed in MSCs under specific experimental conditions. First, Muse cells selectively migrate to damaged tissue after intravenous injection via the S1P-S1PR2 axis, whereas MSCs respond to C-X-C chemokine receptor type 4 and stromal cell-derived factor 1, which are not specific to damaged tissue, and are susceptible to entrapment in the lung [83,118]. The extent to which these differences translate into consistent in vivo target efficiency across models and clinical settings, however, remains to be established. In fact, approximately 14.5% of intravenously administered Muse cells home to the site of damage, while fewer than 1% of intravenously injected MSCs, if any, home to the site of damage [83,119,120]. These estimates may vary depending on model system, methodology, and timing of assessment. Second, due to their targeted migration and homing capacity, clinical trials using Muse cells administered ~1.5 × 10^7^ cells, whereas intravenous administration of MSCs in clinical applications generally requires a ~10-fold higher dose (10–50 × 10^7^) [60,121,122]. Differences in dosing across studies, however, may also reflect variations in study design, indication, and manufacturing protocols rather than intrinsic efficiency alone. Third, Muse cells have been shown to exhibit macrophage-like phagocytic activity and the capacity for pluripotent-like differentiation in experimental settings, thereby contributing to tissue repair. In contrast, MSCs primarily differentiate into osteogenic, chondrogenic, and adipogenic lineages [80,84]. In both cell types, therapeutic effects may also be mediated by paracrine signaling, immunomodulation, and trophic support rather than direct cell replacement alone.

## 7. Discussion: Therapeutic Implications for PD

Pluripotent stem cell-derived dopaminergic precursor therapies have entered early-phase clinical trials, with early studies reporting acceptable safety and evidence of graft survival following transplantation in patients with PD [39,58]. Although these approaches demonstrate feasibility, they continue to face challenges, including intracranial delivery, the need for immunosuppression, genomic quality control, and graft heterogeneity.

Muse cells represent a biologically distinct therapeutic platform. Their ability to home to injured tissue via the S1P–S1PR2 axis, initiate phagocytosis-driven differentiation, and persist in host tissues in preclinical and early clinical studies, including reports without prolonged immunosuppression, suggests a complementary regenerative strategy that may potentially circumvent some logistical and immunologic barriers associated with PSC-based transplantation. Rather than functioning as an adjunct to PSC-derived dopaminergic replacement strategies, Muse cells may represent a mechanistically distinct paradigm that integrates partial cell replacement with microenvironment-responsive repair and may therefore address different translational constraints than lineage-specified progenitor approaches.

Although Muse cells share certain features with MSCs, including surface marker expression and pleiotropic paracrine effects, they differ substantially in several key respects. These include their ability to selectively home to damaged tissues, their reported relatively high therapeutic efficiency in clinical applications, their broader differentiation potential, and their unique phagocytosis-mediated mechanism of tissue repair. Together, these characteristics make Muse cells a promising experimental candidate for regenerative cell replacement therapies, offering potential advantages over conventional MSC-based approaches. Preclinical studies in stroke and ALS models demonstrate durable integration and functional improvement following systemic administration of Muse cells [69,86]. These findings are derived from non-PD disease models, however, and should be interpreted as indirect evidence when considering their relevance to PD. Pathologically, PD is more similar to ALS than ischemic stroke, as both involve progressive neuronal degeneration, whereas ischemic stroke represents an acute injury. While this conceptual similarity is informative, it does not establish translatability of therapeutic effects between diseases. On this basis, systemic administration of donor-derived Muse cells warrants evaluation as a potential treatment for PD. Supporting this concept, intranasally administered Muse cells in a transgenic PD model selectively homed to neurodegenerative regions and exhibited dopaminergic differentiation, as evidenced by marker expression, supporting their potential relevance for chronic neurodegenerative conditions [70]. These findings remain preliminary and do not yet establish functional dopaminergic integration or reconstruction of the nigrostriatal pathway.

Clinical studies also suggest that optimal treatment parameters, including the timing of administration, dosing frequency, and total number of Muse cells, vary depending on disease characteristics. In ischemic stroke, where tissue damage occurs acutely, a single dose of Muse cells appears to be therapeutically effective, consistent with preclinical findings [61,69,107,108]. In contrast, ALS progresses gradually and has remarkably different pathologies from ischemic stroke. Although evaluated in only five ALS patients, repeated monthly infusions (6 total doses) were associated with stabilization or slowing of disease progression [62]. These observations suggest that in chronic diseases such as PD, repeated administration of Muse cells may warrant investigation, although optimal dosing strategies remain to be defined and cannot be inferred directly from other disease models.

## 8. Limitations

In PD, disease duration, baseline motor severity, phenotype, cognitive status, age, and comorbidity burden can strongly influence outcomes, including variability that may complicate interpretation of the treatment effects [5]. Furthermore, positive preclinical results do not always translate into clinical benefit. Behavioral improvements in animal models may reflect neuroprotection, anti-inflammatory effects, trophic support, or nonspecific changes rather than true cell replacement. In addition, animal model studies are often conducted in young transgenic animals, discounting the massive effects of aging processes occurring in humans regardless of disease progression [123]. Consistent with this, the presence of TH-positive cells does not establish functional dopaminergic integration, and further studies are required to demonstrate synaptic connectivity, reconstruction of the nigrostriatal pathway, and sustained functional activity. Although Muse cells are suggested to exert both cell-replacement and pleiotropic effects, the relative contribution of these mechanisms remains unclear, and multiple processes may act in combination.

While potential advantages, such as low immunogenicity and systemic delivery, have been suggested, key uncertainties remain, including the extent of dopaminergic differentiation and functional integration into the substantia nigra, long-term safety and efficacy, and variability across microenvironments. Important practical considerations also remain unresolved, including optimal dosing, treatment frequency and duration, scalability, standardization, and regulatory challenges. Although early studies suggest that intravenous administration of Muse cells may be feasible without HLA matching or prolonged immunosuppression, delayed immunologic risks cannot be excluded, and long-term immune surveillance data are needed, especially in chronic age-related neurodegenerative diseases such as PD.

The studies reviewed are heterogeneous in design, sample size, and statistical rigor, reflecting the early-stage nature of the field. As this is a narrative synthesis, quantitative comparison across studies is limited, and findings should be interpreted in the context of exploratory and hypothesis-generating research rather than definitive effect estimation.

Finally, although Muse cells have been reported to be non-tumorigenic in preclinical studies, these findings are based on limited data, and rare or late adverse events—including ectopic differentiation, abnormal integration, or delayed proliferative risk—cannot yet be excluded. Confirmation through independent replication and larger-scale clinical studies will be essential.

## 9. Conclusions

Over the past three decades, cell replacement therapy has evolved from fetal grafting to increasingly sophisticated PSC-derived dopaminergic progenitor strategies. Early-phase clinical trials using ESC- and iPSC-derived midbrain dopaminergic precursors have demonstrated encouraging safety and feasibility, marking an important milestone in the translational development of regenerative therapies for PD. Nevertheless, significant challenges remain, including the requirement for stereotactic intracranial delivery, the need for prolonged immunosuppression in most allogeneic settings, concerns regarding genomic stability during in vitro expansion, and the risk of cellular heterogeneity or tumorigenicity. The long-term durability of graft function and optimal patient selection criteria also remain to be fully established.

Muse cells represent a biologically distinct endogenous stem cell population that may potentially address several of these limitations. Unlike PSC-derived progenitors, Muse cells can be administered systemically and selectively home to sites of tissue damage via the S1P–S1PR2 axis. Upon arrival, they initiate lineage-specific differentiation through a phagocytosis-driven mechanism and integrate into host tissue in preclinical models without evidence of teratoma formation. Importantly, both preclinical and clinical studies in non-PD conditions have demonstrated that allogeneic Muse cells can be administered in early-phase studies without HLA matching or long-term immunosuppressive therapy, suggesting a potentially favorable immunologic profile for clinical application.

Recent experimental findings now directly support the relevance of Muse cells in Parkinsonian models. Intranasal administration in transgenic PD mice has demonstrated selective homing to degenerating brain regions and, based on marker expression, differentiation toward dopaminergic phenotypes in vivo. Defined in vitro differentiation protocols confirm the intrinsic neurogenic competence of Muse cells without genetic reprogramming, reducing concerns about genomic instability associated with iPSC-based approaches. While these findings remain preclinical and are currently limited to early experimental models, they provide a mechanistic foundation for evaluating Muse cells as a candidate platform for dopaminergic cell replacement.

Further investigation is required to determine whether Muse cell-derived dopaminergic differentiation can translate into sustained functional recovery in established PD models and, ultimately, in clinical trials. Critical next steps include defining optimal cell sources, dosing strategies, delivery routes, and long-term integration dynamics within the degenerating nigrostriatal system. Given their demonstrated safety in early-phase clinical trials for stroke and ALS, Muse cells warrant systematic evaluation in PD as a potential complementary or exploratory regenerative strategy.

In summary, Muse cells offer a mechanistically unique approach to cell-based therapy, integrating targeted homing, phagocytosis-driven differentiation, immune tolerance, and non-tumorigenicity. In PD, current evidence remains limited, indirect, and predominantly preclinical. As the field of regenerative neurology advances, rigorous comparative evaluation of PSC-derived progenitors and endogenous reparative stem cell populations such as Muse cells will be essential for defining the most effective and durable strategy for disease modification in PD.

## Figures and Tables

**Table 1:** Therapeutic stem cell replacement strategies.

Stem Cell Type	Origin and/or Experimental Use	Citations
Human ESCs	Embryonic inner cell mass-derived cell lines	Sonntag, et al. [31], Armstrong, et al. [32]
iPSCs	Fibroblasts or white blood cells	Christiansen and Kirkeby [34], Stoddard-Bennett and Pera [36], Schweitzer, et al. [37], Takahashi [38]
Human iPSC- or ESC-derived dopaminergic progenitor cells	Reprogrammed fibroblasts or white blood cells (iPSCs) or ESCs; differentiated into midbrain dopaminergic neuron precursors; stereotactic intraputaminal transplantation in preclinical and ongoing clinical trials	Clark, et al. [39], Grealish, et al. [40], Takahashi, et al. [41]; Kikuchi, et al. [42], Park, et al. [43], Piao, et al. [44], Sawamoto, et al. [45]
MSCs	Neural crest and mesoderm cells, adult human bone marrow cells differentiated into human dopaminergic neurons	Heris, et al. [35], Trzaska, et al. [46]
MSC-derived Extracellular vesicles (EVs)	Cultured human mesenchymal stem cells with growth factor support	Heris, et al. [35]
Undifferentiated MSCs	6-OHDA rats; improved DA transmission but did not differentiate into dopaminergic neurons	Blandini, et al. [47]

Abbreviations: DA, dopamine; ESCs, embryonic stem cells; iPSCs, induced pluripotent stem cells; MSCs, mesenchymal stem cells; 6-OHDA, 6-hydroxy-dopamine.

## Data Availability

With the exception of the data presented in Figure 4E, which are publicly available in ArrayExpress under accession number E-MTAB-16736, no new data were created or analyzed in this paper.
